# A Guide to Enzymatic Targeted Methylation Sequencing Bioinformatics Workflow: Best Practices

**DOI:** 10.1002/cpz1.70464

**Published:** 2026-10-08

**Authors:** Vitória Rodrigues Guimarães Alves, Sheila T. Nagamatsu, Vanessa Kiyomi Ota, Cibele Edom Bandeira, Janitza L. Montalvo‐Ortiz, Danilo Micali, Ana Carolina Coelho Milani, Ivaldo Silva, Cristiane Seixas Duarte, Andrea Parolin Jackowski, Jonathan Posner, Carolina Muniz Carvalho, Sintia Iole Belangero

**Affiliations:** ^1^ Programa de Pós‐Graduação em Psiquiatria e Psicologia Médica Universidade Federal de São Paulo São Paulo Brasil; ^2^ Department of Psychiatry Universidade Federal de São Paulo São Paulo Brasil; ^3^ LiNC, Laboratory of Integrative Neuroscience Universidade Federal de São Paulo São Paulo Brasil; ^4^ VA Connecticut Healthcare System West Haven CT USA; ^5^ Department of Psychiatry Yale University, School of Medicine New Haven CT USA; ^6^ Programa de Pós‐graduação em Biologia Estrutural e Funcional Universidade Federal de São Paulo São Paulo Brasil; ^7^ Laboratory of Physiological Genomics of Mental Health (PhysioGen Lab) Instituto de Ciências Biomédicas da Universidade de São Paulo São Paulo Brasil; ^8^ Department of Pediatrics Universidade Federal de São Paulo São Paulo Brasil; ^9^ Department of Gynaecology Universidade Federal de São Paulo São Paulo Brasil; ^10^ Department of Applied Health Science, School of Public Health Indiana University Bloomington USA; ^11^ Department of Psychiatry Columbia University Irving Medical Center and New York State Psychiatric Institute New York USA; ^12^ Department of Health & Wellness Design, School of Public Health Indiana University Bloomington USA; ^13^ Department of Psychiatry & Behavioral Sciences Duke University Durham USA; ^14^ Stark Neuroscience Research Institute Indiana University School of Medicine Indianapolis USA; ^15^ These authors contributed equally to this work

**Keywords:** Differentially methylated regions, Epigenetics pipeline, Twist NGS Methylation Detection workflow

## Abstract

We present a best‐practices protocol for targeted DNA methylation sequencing analysis that integrates both bisulfite‐ and enzymatic‐conversion approaches using Twist Bioscience technology. Building on established tools and vendor‐recommended workflows, this protocol provides a standardized, fully reproducible framework that addresses current gaps in the implementation, documentation, and downstream integration of targeted methylation sequencing analyses. The workflow incorporates widely used open‐source tools across two main stages: preprocessing (adapter trimming with Trim Galore and quality control via FastQC/MultiQC) and methylation calling (alignment with BWA‐meth, duplicate marking using Picard, and methylation extraction with MethylDackel). Importantly, we extend existing workflows by introducing a structured third stage for downstream statistical analysis in R, including β‐value calculation, differential methylation analysis with methylKit, principal component analysis, and tiling‐based regional profiling, all embedded in a unified, reproducible pipeline. A key contribution of this protocol is the integration of multi‐stage quality control checkpoints, harmonized parameterization, and explicit reporting of intermediate outputs, enabling transparency and reproducibility across all analytical steps. The modular design allows tool substitution and adaptation to different experimental contexts, including enzyme‐based methylation methods, while maintaining consistency in data processing and interpretation. This guide provides step‐by‐step instructions, troubleshooting strategies, and examples of expected outputs, along with built‐in statistical routines that generate comprehensive results, including methylation matrices, correlation analyses, differentially methylated region annotations, and publication‐ready visualizations. The complete workflow requires approximately 9–27 hr for 10 samples (approximately 134.2 million reads per sample) on Linux systems and assumes moderate proficiency in command‐line environments and R. In summary, this protocol offers a practical, transparent, and reproducible framework that standardizes targeted DNA methylation analysis and facilitates its application across diverse biological and clinical research settings. © 2026 The Author(s). *Current Protocols* published by Wiley Periodicals LLC.

## 1 INTRODUCTION

DNA methylation is an epigenetic modification that plays a fundamental role in regulating gene expression, maintaining genomic stability, and orchestrating cellular differentiation in humans. Aberrant methylation patterns have been implicated in a variety of conditions, including cancer, neurodevelopmental disorders, and environmental exposures (Robertson, 2005). Therefore, accurate, scalable, and reproducible methods for methylation profiling are essential for epigenomic research.

High‐throughput methods for DNA methylation profiling have undergone significant evolution over the past decade. Traditional methodologies such as restriction landmark genomic scanning (RLGS) and DNA adenine methyltransferase identification (DamID) have also contributed to early advances in DNA methylation research (Shu et al., 2020; “Twist Human Methylome Panel | Twist Bioscience,” accessed July 30, 2025). Microarray‐based platforms, such as the latest Illumina MethylationEPIC v2.0 array, offer a cost‐effective, standardized method for interrogating ∼930,000 CpG sites (Pidsley et al., 2016). However, these platforms cover only a fraction of the ∼30 million CpG dinucleotides estimated in the human methylome, limiting discovery beyond predefined genomic regions (Loyfer et al., 2023). Moreover, array‐based approaches do not detect non‑CpG methylation (e.g., CpA, CpT, CpC), which is increasingly recognized as functionally relevant in contexts such as brain tissue and pluripotent stem cells (Jang et al., 2017; Patil et al., 2014; Titcombe et al., 2022). CHG and CHH (where H stands for A, T, or C) methylation is rare in differentiated cells but more common in embryonic stem cells and plants, typically associated with *de novo* methylation maintained by distinct methyltransferases (Smith & Meissner, 2013). Additionally, these techniques often lack resolution and scalability, particularly in non‐model organisms (Vogt, 2022).

In this context, sequencing‐based approaches—particularly targeted bisulfite sequencing (e.g., Reduced Representation Bisulfite Sequencing [RRBS] and Methylation Capture Sequencing [MC–seq])—provide base‐pair resolution and customizable genomic coverage, enabling broader and deeper interrogation of methylation landscapes (Shu et al., 2020). More recently, enzymatic conversion methods have emerged as alternatives to traditional bisulfite sequencing, minimizing DNA damage and improving the detection of methylated cytosines (Vaisvila et al., 2021). One example is the Twist Human Methylome Panel, a capture‐based method that targets over 3.2 million CpG sites across the genome (“Twist Human Methylome Panel | Twist Bioscience,” accessed July 30, 2025). Compared with whole‐genome bisulfite sequencing (WGBS), which offers comprehensive but costly methylome coverage and requires very high sequencing depth, targeted approaches strike a balance among resolution, cost‐effectiveness, and scalability. This makes them particularly suitable for large‐scale studies aimed at detecting subtle epigenetic alterations, such as differentially methylated positions (DMPs) associated with mental health conditions or environmental exposures (Kacmarczyk et al., 2018; Ziller et al., 2015).

Despite the widespread use of sequencing‐based methylation protocols, the bioinformatic analysis remains fragmented and often lacks standardized procedures. Several pipelines have been developed for methylation calling, including MethylDackel, Bismark, and MethPipe, each with strengths and limitations (Krueger & Andrews, 2011; Ryan, 2025; Song et al., 2013). Most of these methods were designed primarily for WGBS and may require substantial computational resources or lack the modular flexibility needed for targeted methylation data.

Recent large‐scale efforts have contributed substantially to the evaluation and standardization of DNA methylation sequencing analyses. For example, Gong et al. (2022) (Gong et al., 2022) provided a comprehensive assessment of WGBS workflows, including practical guidance on available tools and analytical strategies. Similarly, the Sequencing Quality Control 2 (SEQC2) Epigenomics Quality Control (EpiQC) consortium (2021) (Foox et al., 2021) performed a large‐scale benchmarking study to evaluate the performance, reproducibility, and variability of epigenomic profiling methods across platforms and laboratories.

While these studies highlight important sources of technical variability and emphasize the need for robust standards, they primarily focus on benchmarking, cross‐platform comparison, and methodological evaluation. As a result, there remains a lack of practical, end‐to‐end, user‐oriented workflows tailored for targeted methylation sequencing data that integrate preprocessing, quality control, and downstream statistical analysis within a single reproducible framework.

This gap can introduce variability in preprocessing, alignment, and methylation calling strategies, ultimately affecting biological interpretation and reproducibility across studies (Sala et al., 2020). These challenges underscore the need for standardized, transparent, and user‐oriented workflows that support consistent implementation across diverse research settings.

Recognizing these analytical challenges, Twist Bioscience recommended the analysis workflow Analyzing Twist Targeted Methylation Sequencing Data Using the Twist Human Methylome Panel (accessed July 30, 2025), a protocol optimized for target methylation sequencing data and refered here as the Twist NGS Methylation Detection workflow (TNMDW). However, it still lacks detailed step‐by‐step guidance, standardized QC procedures, troubleshooting support, and examples of expected results. Additionally, it does not cover downstream analyses such as differential methylation or data visualization, which are critical for many research applications.

To develop a more user‐friendly workflow, we compiled a best practices guide to enhance clarity, modularity, and reproducibility in the analysis of Twist‐based Panels for DNA methylation data. Here, we use best practices synonymously with fully documented, harmonized parameterization with clear quality control checkpoints at each stage of the workflow; individual parameters were not evaluated against alternatives. Our approach refines and extends the existing TNMDW (“Analyzing Twist Targeted Methylation Sequencing Data Using the Twist Human Methylome Panel | Twist Bioscience,” accessed July 30, 2025) by integrating additional QC steps with differential and statistical analyses. The proposed workflow is customizable and reproducible, ensuring compatibility with a range of experimental contexts. The full guide is publicly available on GitHub and is designed to be computationally efficient, reproducible, and accessible to users with moderate command‐line and R proficiency. We believe this serves as a practical resource for simplifying the analysis of capture‐based methylation sequencing in a variety of research settings.

## 2 PIPELINE WORKFLOW

TNMDW was designed and optimized for short‐read sequencing data, particularly from next‐generation sequencing (NGS) platforms such as Illumina, which produce high‐throughput reads ranging from 50 to 300 base pairs (“Analyzing Twist Targeted Methylation Sequencing Data Using the Twist Human Methylome Panel | Twist Bioscience,” accessed July 30, 2025). Short‐read sequencing is widely used for DNA methylation detection because of its high accuracy, cost‐effectiveness, and suitability for bisulfite‐ or enzyme‐treated DNA (“NGS for Beginners,” accessed March 13, 2025). Following the TNMDW pipeline, we included additional data analysis pipelines for further differential analysis and data visualization (Fig. 1).

**Figure 1:**
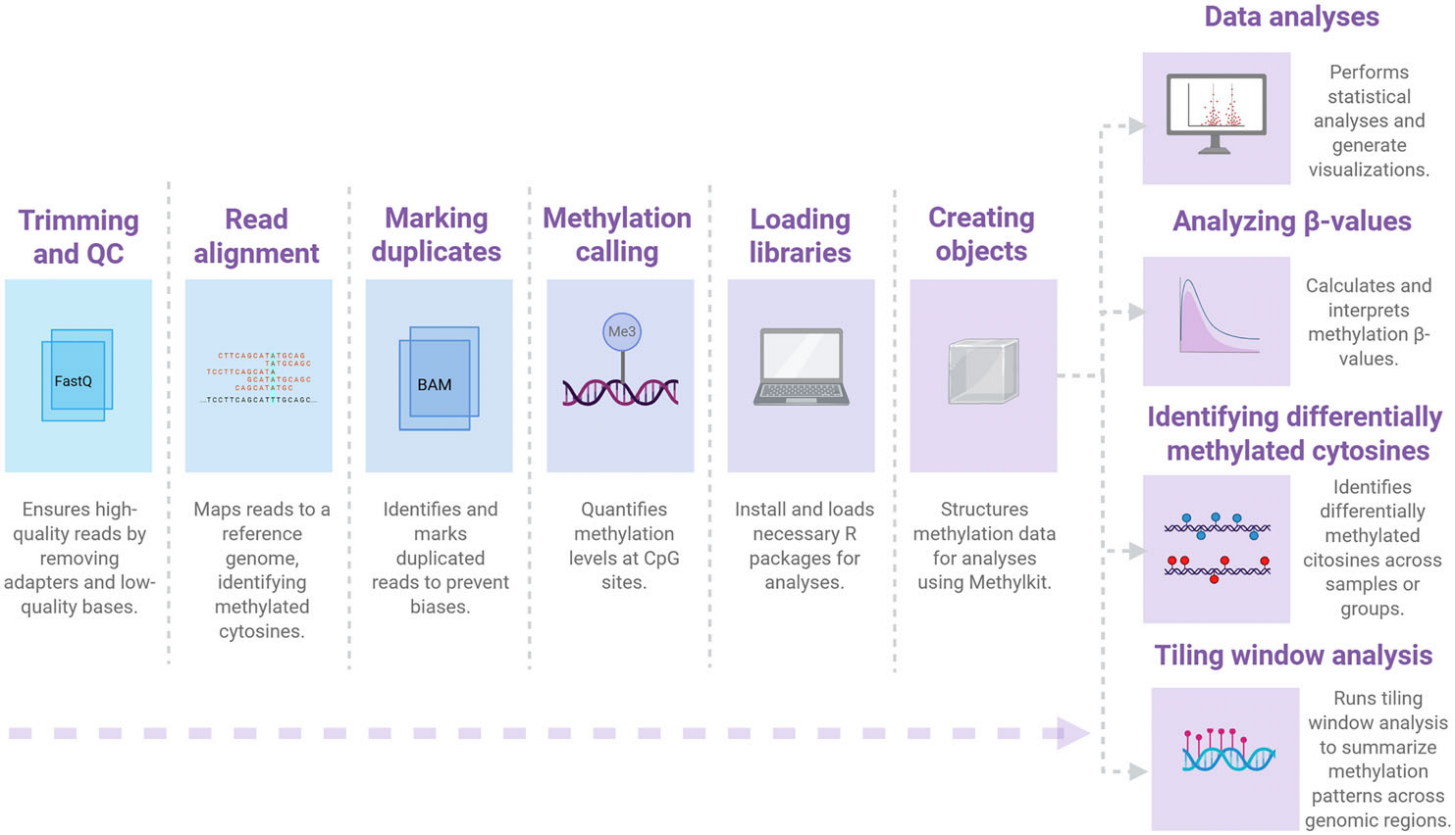
Analysis workflow. Stages performed for the complete data analysis. Figure created with BioRender.com.

At the beginning of each stage, we detail all software and/or R packages used, along with their specific versions, to support common troubleshooting. The environment and scripts used for analysis are also compiled and available on GitHub (access through https://github.com/PGT‐Lab/TNMDW‐best‐practices).

Before starting, users must install the software and R packages listed in Table 1 by following the software and R package tutorials. We strongly recommend that users add the program binary to the PATH or install it in a dedicated conda environment (GATK Best Practices, 2024) so it can be executed from any directory.

**Table 1:** Required tools and download URLs

**Tools and resources**	**Download URL**	**Language**
**Trimming and QC**		
Trim Galore	https://www.bioinformatics.babraham.ac.uk/projects/trim_galore/	Perl
FastQC	https://www.bioinformatics.babraham.ac.uk/projects/fastqc/	Java
MultiQC	https://seqera.io/multiqc/	Python
**Read alignment**		
GRCh38 reference*	https://www.ncbi.nlm.nih.gov/datasets/genome/GCF_000001405.26/	NA
BWA (Li & Durbin, 2009)	https://bio‐bwa.sourceforge.net/	C
Samtools	https://www.htslib.org/	C
**Marking duplicates**		
Java	https://www.java.com/en/	Java
Picard	https://broadinstitute.github.io/picard/	Java
**Methylation calling**		
MethylDackel	https://bio.tools/methyldackel	C
HTSlib	https://www.htslib.org/	C
**Methylation analysis in R**		
RStudio	https://posit.co/	R
BiocManager**	https://cran.r‐project.org/web/packages/BiocManager/index.html	R
methylit**	https://www.bioconductor.org/packages/release/bioc/html/methylKit.html	R

NA: not applicable; *Reference files; **R package.

After installation, users should download the required reference files that best fit the organism being studied. In this guide, we used the human genome GRCh38 (hg38). After downloading the corresponding reference file, it is necessary to create the index files for some of the software required in this pipeline (Table 2).

**Table 2:** Summary of indexing requirements and generation commands

**Tool**	**Index required**	**File extension(s) output**	**Command to generate index**
BWA‐meth	BWA index	.amb, .ann, .bwt, .pac, .sa	bwameth.py index $REF.fa
Samtools	BAM index	.bai	samtools index sample.bam
MethylDackel	FASTA index	.fai	samtools faidx $REF.fa

Table 3 summarizes the input data/files required at each stage of the pipeline, along with the respective output data/files. Example input datasets can be obtained from public repositories such as Gene Expression Omnibus (GEO).

**Table 3:** Input and Output of each stage

**Stages**	**Input**	**Output**
**Best practices for the TNMDW workflow (Stages 1**–**4)**		
Trimming and QC	Raw sequence reads in .fastq.gz	.fastq.gz; .html
Read alignment	Trimmed sequence reads in .fastq.gz	.bam
Marking duplicates	Aligned and sorted sequence reads in .bam	.bam; .txt; .pdf
Methylation calling	Sequence reads marked for duplicates in .bam	.svg; .bedGraph; .txt; .methylKit
**Methylation analysis in R (Stages 5**–**8)**		
Loading libraries and data	.methylKit sequence file	object with case and control lists
Creating objects for data analysis	Object with case and control lists	i) methRead object ii) CpG coverage filtered object iii) CpGs present in at least 1 sample object iv) methylBase object v) Data frame
Data analysis	methRead object (Creating objects for data analysis output)	i) Graphs ii) Hyper‐ and hypomethylated region files
Analyzing methylation β‐values	methylBase object (Creating objects for data analysis output)	.csv and .png
Identifying differentially methylated cytosines	CpGs present in at least 1 sample object (Creating objects for data analysis output)	Matrix
Tiling window analysis	methRead object (Creating objects for data analys output)	methylBase object

### 2.1 Best practices for the TNMDW workflow

For the sections below, we have compiled the TNMDW pipeline (“Analyzing Twist Targeted Methylation Sequencing Data Using the Twist Human Methylome Panel | Twist Bioscience,” accessed July 30, 2025), from trimming through methylation calling, with additional QC steps for data verification. We have enumerated the command lines in sequential logical order for better understanding and visualization. The optional commands for downsampling reads from the original protocol were excluded in our guide to preserve the full depth of sequencing coverage, which is critical for maximizing sensitivity and accuracy in downstream methylation analyses (“Analyzing Twist Targeted Methylation Sequencing Data Using the Twist Human Methylome Panel | Twist Bioscience,” accessed July 30, 2025).

#### 2.1.1 Trimming and Quality Control

Raw sequencing data often contains adapter sequences, low‐quality bases, and artifacts that can impact downstream analysis. Evaluating QC and performing trimming ensure high‐quality reads, improving alignment accuracy and methylation calling. A post‐trimming QC check is also performed to assess read quality, GC content, sequence duplication, and overall data integrity.

**Input files**: Raw sequence reads in fastq.gz format.

**Adaptations**: The parameters “threads” and “core” must be adjusted according to the available computational power. Besides, the user can choose the “outdir” and “output_dir” where the outputs will be saved.


#------------------
#trim_galore v0.6.7 - powered by Cutadapt
#FastQC v0.11.9
#multiqc v1.22
#------------------

1. Run the fastqc command pre‐trimming to verify the quality of the fastq.gz files.


fastqc --noextract --threads 8 *fq.gz --outdir [PATH]

2. Trim adapters from sequence read files. The parameter “cores” can be adjusted to match the available computational power.


trim_galore --gzip --cores 2 --output_dir [PATH] --2colour 20 --paired "$FQ1" "$FQ2"

3. Rerun the fastqc command to check whether all adapter content has been removed.


fastqc --noextract --threads 8 *fq.gz --outdir [PATH]

4. OPTIONAL: Run multiqc to combine all .html files created in Step 1 or 3, eliminating the need to manually verify multiple files individually. The parameter “.” specifies the current directory (containing the .html files) as the starting point for the search:


multiqc. --outdir [PATH]

#### 2.1.2 Read alignment

The goal of an alignment is to map the sequenced reads to a reference genome. In our case, we used the human genome, hg38 (for guidance and best practices on selecting the proper reference genome, visit: https://www.ncbi.nlm.nih.gov/datasets/docs/v2/policies‐annotation/genome‐processing/reference‐selection/). This stage identifies regions in the genome similar to each read and places them in the best position.

For methylated cytosines (C), we need an additional step during alignment to ensure reads are paired correctly, since they are converted to uracils during the conversion step. To ensure accurate alignment of bisulfite‐ or enzymatically converted reads, bwameth aligns bisulfite‐ or enzymatically converted reads to multiple in silico–converted versions of the reference genome (e.g., C→T and G→A). This approach accommodates strand‐specific conversion patterns and allows detection of both methylated and unmethylated cytosines (Li & Durbin, 2009).

To assist users, Figure 2 shows a visual representation of the major issues found during the alignment and marking duplicates steps (for further troubleshooting directions, consult Tables 4 and 6).

**Figure 2:**
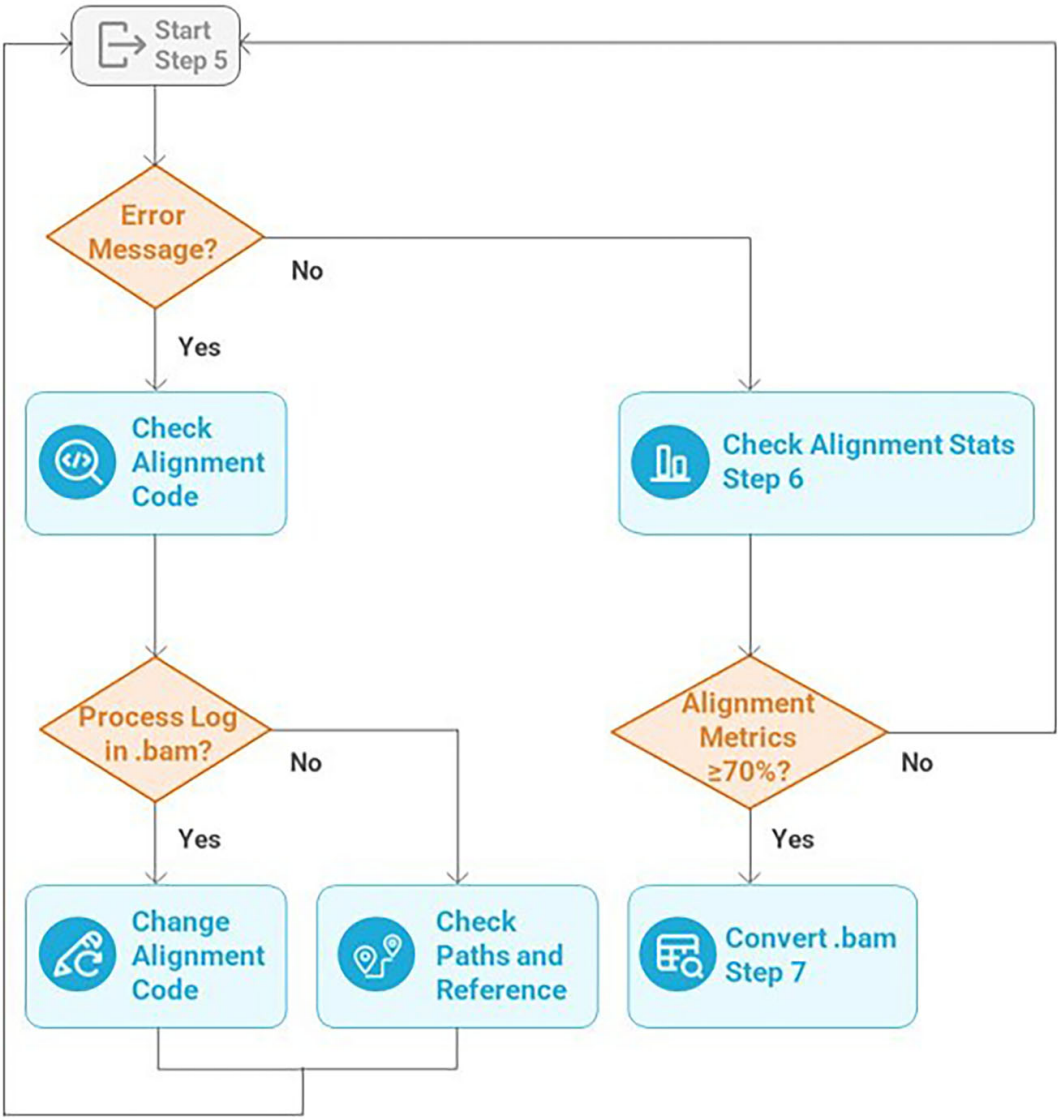
Main troubleshooting steps. Workflow of the major problems during read alignment and duplicate marking (steps 6–15) and possible solutions. Image generated with the aid of Napkin AI (https://www.napkin.ai/pt/). The final layout and review were carried out by the authors.

**Table 4:** Important alignment metrics

**Metric**	**Definition**	**Importance**	**Integrity (%)**
Mapped reads	Reads that are successfully aligned to the reference genome	Indicates overall read quality and compatibility with the reference	If this number changes at any point, it means some aligned reads were lost—a serious issue
Primary mapped	Reads with a single main alignment (excluding secondary/supplementary)	Reflects unambiguous mapping; fewer multi‐mapped reads = better alignment quality	Changes here suggest improper filtering or data corruption during file manipulation
Properly paired	Paired‐end reads mapped with correct orientation and expected insert size	Shows whether sequencing and mapping of read pairs are accurate and consistent	Differences indicate read pairs were split or mishandled, which can affect downstream analyses like variant calling

**Input files**: Trimmed sequence reads in .fastq or .fastq.gz format.

**Adaptations**: The parameters “threads” and “@” must be adjusted based on available computational power.

Troubleshooting: step 6.


#---------
#BWA (Burrows2-Wheeler Aligner) v0.7.18
#samtools v1.20
#---------

5. Align both fq.gz files of each sample to the Reference genome (previously downloaded and indexed—please see command in Table 2):
**IMPORTANT NOTE**: When running this code for multiple samples, ensure that all variables (*i.e*., tID, tSM, and the $FQ1 and $FQ2 files) refer to the same sample. This example assumes paired‐end sequencing, where two fastq files ($FQ1 and $FQ2) contain forward and reverse reads, respectively. Paired‐end data typically improves mapping accuracy and methylation calling.
  If using single‐end data, provide only one fastq file. The rest of the command remains unchanged, but note that alignment performance and downstream resolution may differ from those in paired‐end mode.


bwameth.py --threads 5 \
--reference $REF \
--read-group '@RG\tID:XX\tPL:XX\tSM:XX' \
## ID=sample ID; PL=sequencing platform used; SM=sample name/number ##
$FQ1 $FQ2 > some.bam

6. Check integrity and sample stats—this step was added to the original workflow to verify the quality of the output:
**IMPORTANT NOTE**: The metrics generated in this step offer critical information about your alignment. It indicates the percentages of “mapped,” “primary mapped,” and “properly paired” reads, which reflect the quality of the alignment (Table 4). Usually, reads with ≥70% are considered of good quality (Li & Durbin, 2009). However, users are encouraged to consider the sequences and organisms used in the analysis, as those percentages may vary accordingly. A low percentage may also indicate problems with the reference genome and contamination.


samtools flagstat some.bam

7. Convert .bam to use with other tools—this step was added to ensure that the .bam file is correctly formatted and compressed for downstream compatibility and efficiency:


samtools view -@ 3 -b some.bam > some_converted.bam

8. Re‐check integrity and sample stats—this step was added to verify the quality of the output:
**IMPORTANT NOTE**: Make sure the sample statistics in Table 4 have not changed after conversion.


samtools flagstat some_converted.bam

9. Sort sample:


samtools sort -O bam -o some_sorted.bam some_converted.bam

10. Check integrity and sample stats—this step was added to verify the quality of the output:
**IMPORTANT NOTE**: Ensure that the sample statistics listed in Table 4 have not changed after sorting.


samtools flagstat some_sorted.bam

11. Index alignment:


samtools index some_sorted.bam

#### 2.1.3 Marking duplicates

During library preparation and sequencing, duplicate reads can arise from biological factors or technical artifacts, artificially inflating coverage and introducing biases in read counts and, consequently, methylation quantification. Thus, identifying and marking duplicate reads prevents these errors by preserving information and preventing them from contributing to the analysis.

**Input files**: Aligned and sorted sequence reads in .bam format.

**Adaptations**: The parameter “@” must be adjusted according to the available computational power.

Troubleshooting: step 15.


#---------
#java v19.0.2
#Picard v2.7.3
#---------

12. Mark the duplicated reads in each sample — following the TNMDW, the optical pixel distance should be changed according to the type of flow cell (i.e., random or patterned): random = 100 and patterned = 2500, consistent with GATK best practices (“Pixel Distance for MarkDuplicates (Picard),” 2024). Although this pipeline is traditionally associated with variant calling workflows, its recommendations for duplicate marking are broadly applicable across NGS applications, including DNA methylation studies (Broad Institute | Picard, accessed April 08, 2025):

java -jar $PATH/picard.jar MarkDuplicates -I some_sorted.bam -O some_sorted.markdup.bam -R $REF.fa -M some_sorted.picard_markdup_raw_metrics --CREATE_INDEX false --MAX_RECORDS_IN_RAM 1000 --SORTING_COLLECTION_SIZE_RATIO 0.15 --ASSUME_SORT_ORDER coordinate --OPTICAL_DUPLICATE_PIXEL_DISTANCE 2500

13. Check integrity and sample stats — this step was added to verify the quality of the output:
**IMPORTANT NOTE**: Make sure the sample stats mentioned in Table 4 have not changed, and that “duplicates” and “primary duplicates” are marked in the sequence, as shown in the same output.


samtools flagstat some_sorted.markdup.bam

14. Create index:


samtools index -@ 4 some_sorted.markdup.bam

15. Check insert size metrics — this step was added to ensure proper read alignment
**IMPORTANT NOTE**: The graph created (Fig. 4) in this step should resemble an inverted U‐shaped distribution. If the graph shows multiple vertical lines, it may result from an incorrect read alignment.


java -jar $PATH/picard.jar CollectInsertSizeMetrics \
I=some_sorted.markdup.bam \
O=some_sorted.markdup_size_metrics.txt \
H=some_sorted.markdup_size_histogram.pdf \
M=0.5

16. OPTIONAL: Collect alignment summary metrics—this step was added to the original workflow to detect technical artifacts that confound methylation analysis. Always use IS_BISULFITE_SEQUENCED=true for any conversion‐based methylation analysis (bisulfite or enzymatic) to prevent false inflation of mismatch rates, incorrect strand bias calculations, and misinterpretation of adapter contamination.


java -jar $PATH/picard.jar CollectAlignmentSummaryMetrics \
R=$REF.fa \
I=some_sorted.markdup.bam \
O=some_sorted.markdup.txt \
HISTOGRAM_FILE=some_sorted.markdup_histogram.pdf \
IS_BISULFITE_SEQUENCED=true

17. OPTIONAL: Collect GC bias metrics — this step was added to the original workflow for detecting technical artifacts that confound methylation analysis, particularly in CpG islands:


java -jar $PATH/picard.jar CollectGcBiasMetrics \
I=some_sorted.markdup.bam \
O=some_sorted.markdup_gc_bias_metrics.txt \
CHART=some_sorted.markdup_gc_bias_metrics.pdf \
S=some_sorted.markdup_summary_metrics.txt \
R=$REF.fa

#### 2.1.4 Methylation calling

Methylation calling estimates cytosine methylation levels by distinguishing between converted (unmethylated) and unconverted (methylated) cytosines. The percentage of methylation at CpG, CHH, and CHG sites is quantified by calculating the ratio of methylated cytosines (read as C) to the total number of cytosines at each position (C + T), based on aligned sequencing reads (Ryan, 2025). Further, quality filters are applied to exclude low‐confidence methylation calls.

**Input files**: Sequence reads marked as duplicates in .bam format.

**Adaptations**: The parameter “@” must be adjusted based on available computational power.

Troubleshooting: step 18.


#---------
#MethylDackel v0.6.1
#HTSlib v1.20
#---------

18. Check bias and clean reads:
**IMPORTANT NOTE**: OT (Original Top) and OB (Original Bottom) refer to reads that map directly to the top or bottom strands of the original DNA, as MethylDackel classifies reads based on their strand of origin. Graphs generated in this step must have a number other than 0 in them (*e.g*., –OT 0,0,0,146; –OB 3,0,5,0). If all numbers are shown as 0, there was an issue with either the alignment or the duplicated marking steps (Ryan, 2025).

MethylDackel mbias $REF.fa --@ 3 some_sorted.markdup.bam some_methylome

19. Add filters and generate CpG calls (the OT and OB values are derived from the.svg output graphs generated in the previous step):


MethylDackel extract --minDepth 10 --maxVariantFrac 0.25 --OT X,X,X,X --OB X,X,X,X --mergeContext $REF.fa some_sorted.markdup.bam -o some_methylome

20. OPTIONAL: Generate methylKit file (only needed if methylKit will be used for further analysis):

MethylDackel extract --minDepth 10 -@ 3 --maxVariantFrac 0.25 --methylKit –OT X,X,X,X --OB X,X,X,X $REF.fa some_sorted.markdup.bam -o some_methylome_report

21. Apply filters and generate cytosine‐level reports:


MethylDackel extract --minDepth 10 -@ 3 --maxVariantFrac 0.25 --OT X,X,X,X --OB X,X,X,X --cytosine_report --CHH --CHG $REF.fa some_sorted.markdup.bam -o some_methylome_report

### 2.2 Methylation analysis in R

The following steps, implemented in the RStudio environment (download URL in Table 1), were integrated into the TNMDW to support differential methylation analysis. These suggested additions provide processed outputs and visualizations that are publication‐ready, facilitating data interpretation and reporting. The code was extracted from the official methylKit user guide to ensure compatibility with the recommended practices for bisulfite and enzymatic sequencing data analysis presented (Akalin et al., 2012; Gentleman et al., 2004).

#### 2.2.1 Loading libraries and data

Before performing methylation analysis in R, it is essential to load the necessary packages and import the data. This step ensures that all dependencies are installed and the input files are properly structured for downstream analysis.

**Input files**: MethylKit sequence file generated in the previous step (*Methylation calling*).

**Adaptations**: The following parameters must be adjusted: path to the folder users want to set as working directory (“PATH”), path to the first case/control sample file (“PATH_1”), path to the second case/control sample file (“PATH_2”), case/control sample ID number X (“XX”), and case/control sample ID number Y (“YY”).

22. Install all required libraries:


if (!requireNamespace("BiocManager", quietly = TRUE))
install.packages("BiocManager")
install.packages("R.oo")
BiocManager::install("methylKit")
BiocManager::install("genomation")

23. Load the libraries:


library(methylKit) #Processes and analyzes DNA methylation data from bisulfite sequencing
library(genomation) #Handles and visualizes genomic annotations and epigenetic data
library(R.oo) #Enables object-oriented programming in R
library(dplyr) #Facilitates efficient data manipulation and transformation

24. Set working directory:

setwd("PATH")

25. Load the files of “case” subjects:


case_files <- list ("PATH_1",
"PATH_2", ...)

26. Enter the “case” subject IDs:


case_id <- list ("XX", "YY", ...)

27. Load the files of “ controls” subjects:

control_files <- list ("PATH_1",
"PATH_2", ...)

28. Enter the “controls” subject IDs:

control_id <- list ("XX", "YY", ...)

29. Combine both ID lists:

sample_id <- c(case_id, control_id)

30. Create treatment vector (1 for cases and 0 for controls):

case <- rep(1, length(case_files))
control <- rep(0, length(control_files))
treatment <- c(case, control)

31. Combine case and control files list:

files <- c(case_files, control_files)

#### 2.2.2 Creating object for data analysis

This stage involves loading and structuring the methylation data for further analysis. MethylKit package is used to read, filter, and prepare the data, ensuring that high‐quality CpG sites are retained (Akalin et al., 2012; Gentleman et al., 2004).

**Input files**: files, sample_id and treatment objects created in steps 29‐31.

32. Create methRead object — the parameter “mincov = 10” can be modified according to users' needs:

myobj=methRead(files,
sample.id=sample_id,
assembly="hg38", # change if your Ref is other than hg38
treatment=treatment,
context="CpG", # use CHG/CHH for non-CpGs analyses
mincov = 10 # keep reads with more than 10×
)

33. Filtering samples by CpG coverage — The parameters “lo.count=10, lo.perc=NULL” are used to ensure consistent filtering across samples, while “hi.perc=99.9” is used to remove the 0.1% of CpGs with the highest coverage (e.g., outliers); all can be changed according to the desired statistical robustness:

filtered.myobj=filterByCoverage(myobj, lo.count=10, lo.perc=NULL,
hi.count=NULL, hi.perc=99.9)

34. Filtering for CpGs present in at least 1 sample. This object will be used later for further steps:


meth.min=unite(filtered.myobj, min.per.group=1L)

35. Combine all samples — converting object to methylBase for β‐value analysis:

methyl_base <- unite(filtered.myobj, destrand = FALSE)

36. Extract data to a data frame:

methyl_base_data <- getData(methyl_base)

#### 2.2.3 Data analysis

In this study, we conduct a statistical and exploratory analysis of the processed methylation data. Our aim is to assess read coverage, identify methylation patterns, evaluate sample correlations, and perform dimensionality reduction techniques such as clustering and principal component analysis (PCA). This analysis provides a comprehensive overview of data quality, variability, and key methylation differences, forming the foundation for further downstream biological interpretations.

**Input files**: myobj object created at the beginning of the previous step.

37. Calculate CpG read coverage — This ensures sufficient and consistent coverage for reliable methylation calling and helps identify potential low‐quality samples or coverage outliers.

for (i in 1:length(myobj)) {
cat("Sample statistics", i, ":", myobj[[i]]@sample.id, "\n")
getCoverageStats(myobj[[i]], plot=TRUE)
}

38. Calculate methylation percentage distribution — This aids in detecting potential biases or batch effects and in verifying that the biological range of methylation is preserved.

for (i in 1:length(myobj)) {
cat("Sample statistics", i, ":", myobj[[i]]@sample.id, "\n")
getMethylationStats(myobj[[i]], plot=T, both.strands=FALSE)
}

39. Make a correlation graph to measure the pairwise correlation of methylation profiles between samples:

getCorrelation(meth.min, plot=TRUE)

40. Generate a dendrogram — This unsupervised clustering helps detect outliers, assess replicate grouping, and reveal underlying biological or experimental structure.

clusterSamples(meth.min, dist="correlation", method="ward", plot=TRUE)

41. Run the PCA — This analysis helps detect major sources of variance, uncover sample clustering patterns, and evaluate the influence of experimental conditions or batch effects.

PCASamples(meth.min, screeplot=TRUE)

42. Generating the PCA graph:

PCASamples(meth.min)

#### 2.2.4 Analyzing methylation β‐values

To facilitate cross‐platform comparisons and external visualization, we computed β‐values from the methylKit output using a custom R script. The β‐value metric is more commonly used in array‐based studies and enables standardized representation across datasets. It is calculated as follows (Du et al., 2010):

$$\beta =\frac{numCs}{numCs+numTs}$$

We therefore implemented a script to extract the raw counts (*numCs* and *numTs*) from the methylBase object and calculate β‐values for each sample, while avoiding division by zero by assigning NA to undefined values. A summary table was generated with descriptive statistics (minimum, quartiles, mean, maximum) for each sample, followed by visual representations using boxplots and scatterplots to assess β‐value distribution across all samples. This approach was added to complement MethylKit outputs with publication‐ready visual summaries.

**Input files**: methyl_base_data object created in the “*Creating object for analysis*” stage.

43. Create an empty data frame with the same row number as methyl_base_data:

beta_values <- data.frame(matrix(ncol = 0, nrow = nrow(methyl_base_data)))

44. Calculate β‐values:

for (i in 1:20) { # Adjust sample number as needed
numCs <- methyl_base_data[[paste0("numCs", i)]]
numTs <- methyl_base_data[[paste0("numTs", i)]]
# Prevent Division by 0
beta <- ifelse((numCs + numTs) > 0, numCs / (numCs + numTs), NA)
# Add column to df
beta_values[[paste0("beta", i)]] <- beta
}

Steps 45 to 49 create a table of all the β‐value data, convert it to a data frame, and export it as a .csv file that can be used by other programs and software.

45. Summarize all β‐values variables:

summary_table <- sapply(beta_values, summary)

46. Convert table to a data frame:

summary_table <- as.data.frame(t(summary_table))

47. Add column names:

colnames(summary_table) <- c("Min", "1st_Qu", "Median", "Mean", "3rd_Qu", "Max", "NA")

48. Rename rows with sample IDs:

rownames(summary_table) <- sample_id

49. Export data frame to a .csv file:

write.csv(summary_table, file = "summary_table.csv", row.names = TRUE)

50. Creating a boxplot graph for all samples:

boxplot(beta_values,
main = "Sample Beta-values",
xlab = "Samples",
ylab = "Beta-values",
col = "skyblue",
las = 2) # Rotate sample names for better visualization

51. Creating a vector with all mean values — needed for generating a scatterplot type of graph:

mean <- summary_table$Mean

52. Creating a scatterplot graph:

plot(1:length(mean), mean,
type = "p",   # just dots
pch = 16,   # full circle
col = "blue", # color
xlab = "Samples", # X axis
ylab = "Mean Beta-values", # Y axis
main = "Mean Beta-values Dispersion", # Title
xaxt = "n")   # Hide X axis

53. Adding sample IDs to the x‐axis of the scatterplot graph:

axis(1, at = 1:length(mean), labels = rownames(summary_table), las = 2)

#### 2.2.5 Identifying differentially methylated cytosines

This stage aims to detect differentially methylated cytosines (DMCs) between case and control groups (manually defined by the user in steps 26 and 28). Identifying DMCs is crucial for understanding epigenetic modifications associated with environmental exposures, diseases, or developmental processes.

The function used (calculateDiffMeth()) estimates DMCs between the previously defined groups (case vs. control) by applying logistic regression. It compares the proportions of methylated and unmethylated reads across samples, accounting for biological variation and read coverage. The model tests whether methylation levels differ significantly between groups at each CpG site (Akalin et al., 2012).

**Input files**: meth.min object created in the “*Creating object for analysis*” (step 34).

54. Calculating DMCs:

myDiff=calculateDiffMeth(meth.min)

55. Creating a matrix containing percent methylation values:

perc.meth=percMethylation(meth.min)

56. Filtering hyper‐ and hypomethylated regions into two different files:


myDiff.hyper=getMethylDiff(myDiff, difference=2, qvalue=0.01, type="hyper")
myDiff.hypo=getMethylDiff(myDiff, difference=2, qvalue=0.01, type="hypo")

#### 2.2.6 Tiling window analysis

This stage is a segmentation approach used to analyze DNA methylation across genomic regions rather than at individual CpG sites. This method improves statistical power and biological relevance by identifying DMCs within fixed‐length genomic windows. By aggregating CpG sites into windows, this analysis provides a more stable and biologically meaningful representation of methylation changes, making it particularly useful for identifying functionally relevant epigenetic modifications.

**Input files**: files list created in Step 31.

**Adaptations**: The parameters “mincov” and “difference” can be adjusted according to the study design and the desired statistical sensitivity.

57. Creating an object. We set “mincov = 3” to retain CpG sites with moderate coverage, allowing broader inclusion of sites during aggregation. The parameter “mincov = 3” can be modified according to users' needs:


myobj_lowCov=methRead(files,
sample.id=sample_id,
assembly="hg38",
treatment=treatment,
context="CpG",
mincov = 3 # keep reads with more than 3×
)

58. Creating tiles. Tiling was performed using 1,000‐bp windows with a requirement of at least 10 covered CpGs per window (cov.bases = 10), ensuring statistical robustness at the regional level despite relaxed per‐site thresholds.

tiles = tileMethylCounts(myobj_lowCov, win.size=1000, step.size=1000, cov.bases = 10)

59. Merge all samples to one table by using base‐pair locations that are covered in all samples:

meth_low = methylKit::unite(tiles, destrand = FALSE)

60. Calculating differential methylation. Differential methylation analysis was performed on the unified tile‐based methylation object:

tile_diff = calculateDiffMeth(meth_low)

61. Filtering hyper‐ and hypomethylated tiles into two different files — The parameter “difference” was set to 10 to select tiles showing at least a 10% absolute methylation difference between groups:

tile_diff_hyper = getMethylDiff(tile_diff, difference = 10, qvalue = 0.01, type = "hyper")
tile_diff_hypo = getMethylDiff(tile_diff, difference = 10, qvalue = 0.01, type = "hypo")

## 3 TIMING

To help researchers anticipate computational resources and time requirements, Table 5 outlines the estimated duration for each stage of the complete analysis pipeline, from initial preprocessing to downstream methylation analyses in R.

**Table 5:** Estimated duration of the complete analysis

Stages	Estimated time (10 samples*)	Notes
**Command‐line pre‐processing**		
**Trimming and Quality Control** Steps 1–4	30 min–2 hr	Depends on read length, sequencing depth, and number of threads
**Read alignment** Steps 5–11	4–12 hr	Main computational bottleneck; highly dependent on sequencing depth
**Marking duplicates** Steps 12–17	2–5 hr	Influenced by sample complexity and read count
**Methylation calling** Steps 18–21	30 min–2 hr	Varies with depth and genome size
**Total time estimates**	7–21 hr	Varies depending on read length, coverage, and computational setup
**Methylation analysis in R**		
**Loading libraries and data** Steps 22–31	3–10 min	One‐time setup; depends on the number of sample reads
**Creating object for analysis** Steps 32–36	20–50 min	Depends on number of CpGs and coverage thresholds
**Data analysis** Steps 37–42	30 min–2 hr	Increases with sample size and CpG density
**Analyzing methylation β‐values** Steps 43–53	30–60 min	Fast step; parallelizable if needed
**Identifying differentially methylated cytosines** Steps 54–56	30–60 min	Influenced by number of CpGs
**Tiling window analysis** Steps 57–61	30–60 min	Influenced by number of tiles and coverage filtering
**Total time estimates**	2–6 hr	Varies depending on read length, coverage, and computational setup

All durations may vary depending on read length, coverage, and computational setup. Parallelization is supported in most steps, and batch‐wise processing is recommended for large cohorts to manage memory usage efficiently. The hardware specifications used for the comparisons are described in Section 7.1, “Equipment.” *Each sample had approximately 134.2M reads.

## 4 TROUBLESHOOTING

To assist users in resolving potential issues encountered during the analysis, Table 6 summarizes common problems, likely causes, and recommended solutions for each step of the pipeline. This section is intended to improve reproducibility and minimize technical difficulties throughout the workflow.

**Table 6:** Troubleshooting steps

Step	Problem	Possible Reason	Solution
6	After running the code, users may get this error message: fail to read the header from “some.bam”	The process log has been saved to the .bam file rather than the sequence itself	Run: head ‐n 10 some.bam If the exit starts with “Found BWA MEM index,” then the process log has been saved—users need to change the alignment code to the following: bwameth.py –threads 5 \ –reference $REF \ –read‐group ‘@RG\tID:XX\tPL:XX\tSM:XX’ \ $FQ1 $FQ2 2> some.log |\ samtools view ‐bS ‐o [PATH]/some.bam
	“mapped,” “primary mapped,” and “properly paired” percentages are under 90% or show N/A.	The alignment has not been done properly	Make sure that: (i) all paths are entered correctly; (ii) the right reference is being used; (iii) the reference index has been created correctly and is in the same folder as the reference sequence. After ensuring everything is set up properly, run the code again.
15	The graph created has various vertical lines rather than an inverted U shape	The alignment has not been done properly	Check the troubleshooting solutions for Step 6; users may need to redo the alignment
18	OT and OB graphs generated have all numbers as 0	There was an issue with either the alignment or the duplicated marking steps.	If the alignment stats are over 90%, then users may need to redo Step 12 and mark the duplicates again.

## 5 EXPECTED QC Results

This section presents key output metrics used for quality control across various stages of the workflow. Ensuring data quality at each step is essential for the reliability and accuracy of downstream analyses.

After removing adapters from sequence reads and running the fastqc and multiqc commands, users should expect results similar to those shown in Figure 3. These graphs provide an overview of sequencing quality, including per‐base sequence quality scores, sequence duplication levels, and GC content distribution.

**Figure 3:**
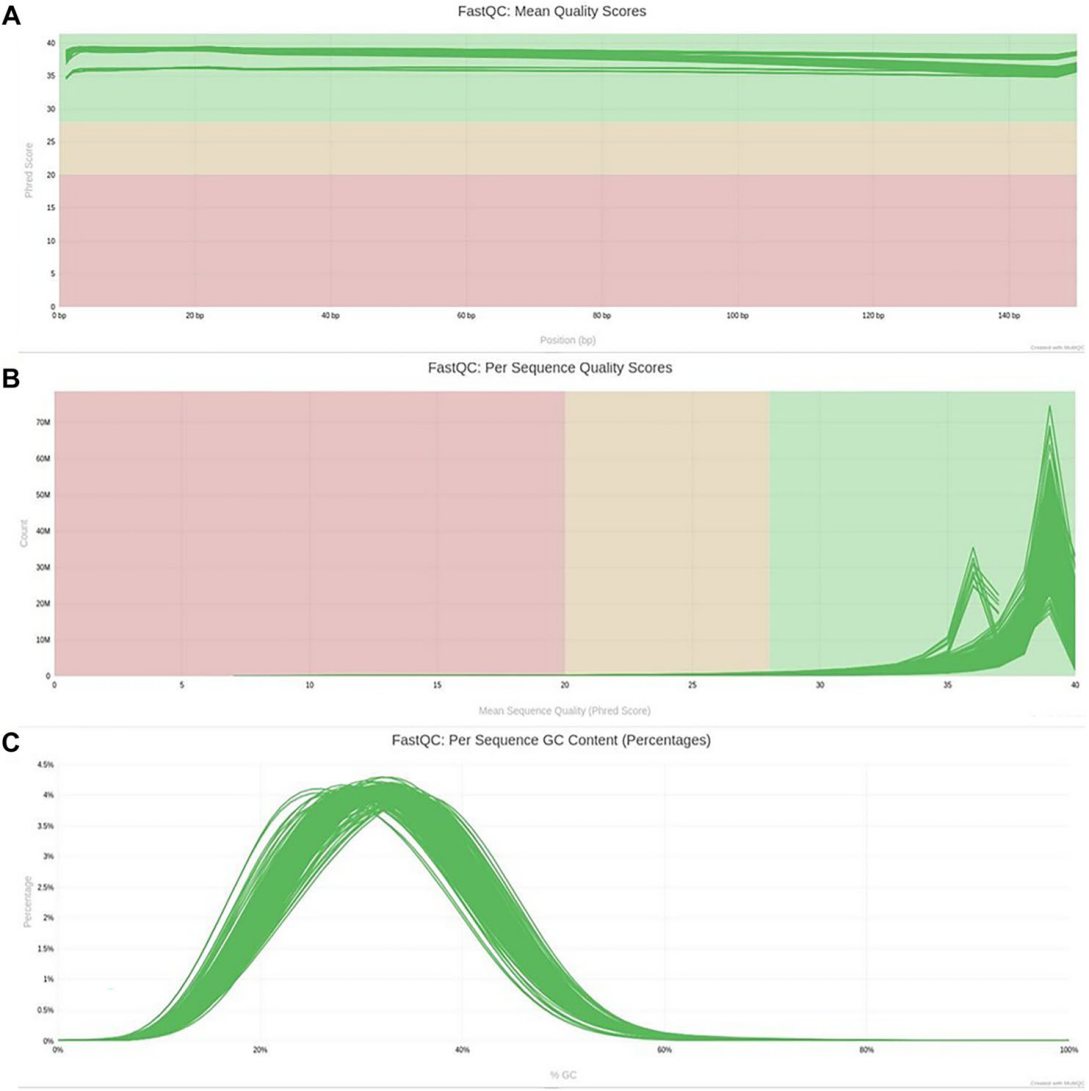
Quality control graphs generated with FastQC (Step 2). From top to bottom: 3a Mean Quality Scores; 3b Per‐Sequence Quality Scores; and 3c Per‐Sequence GC Content (Percentages). Dark green lines represent each sample sequence read.

Ideally, all samples should fall within the green area of the top two graphs in Figure 3, indicating high‐quality reads suitable for further analysis (Phred score > 28). The bottom graph represents the GC content of each sample, which should follow an inverted U‐shaped curve within a GC range of 20% to 40%. Any deviations, such as a shifted or flattened curve, may indicate contamination or sequencing bias and require further investigation before proceeding.

Once the initial QC is passed, users proceed with read alignment (Steps 5–11) and duplicate marking (Steps 12–17). If all commands execute without errors, the duplication summary graph generated in Step 15 should resemble Figure 4. A moderate level of duplication is expected in bisulfite sequencing data, but excessive duplication may suggest PCR bias or over‐sequencing of the same molecules, which could affect downstream analyses.

**Figure 4:**
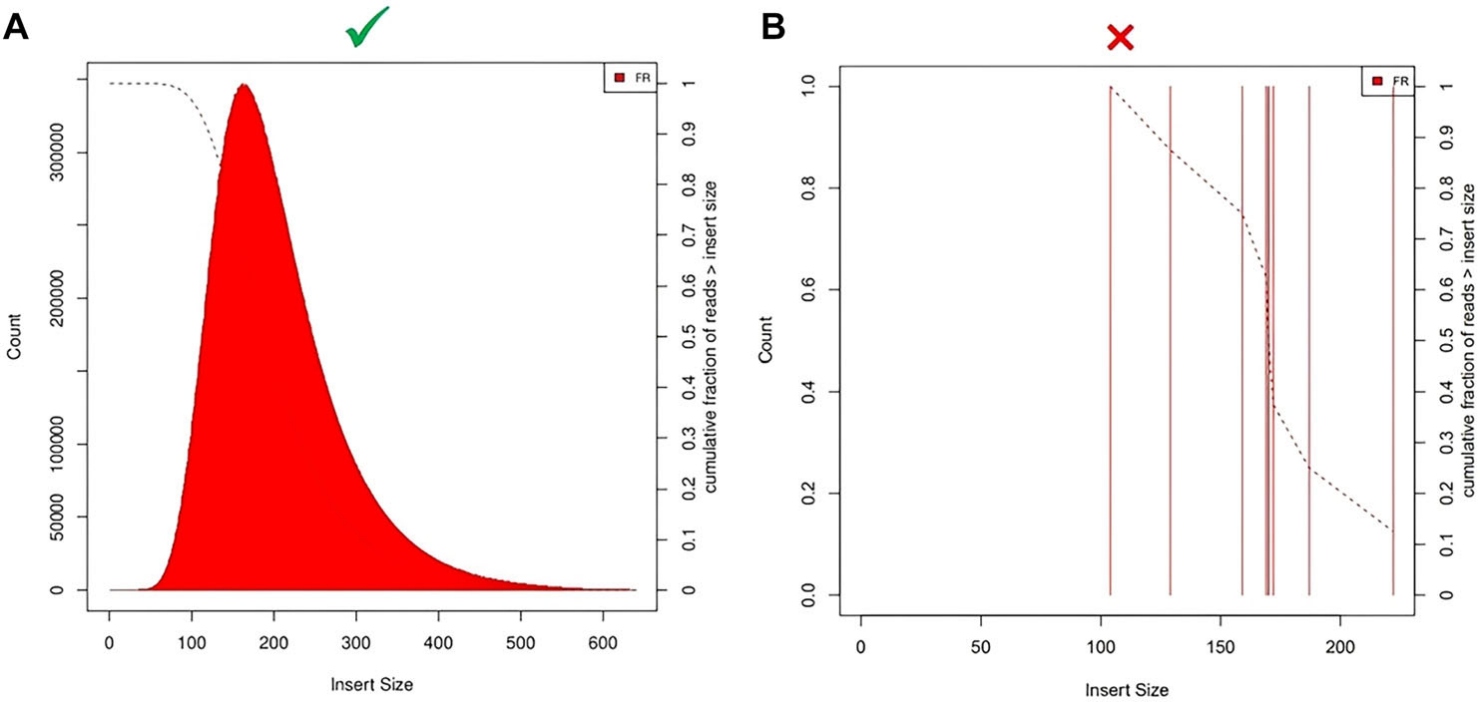
Insert size metrics. The ideal graph result (4a) and the incorrect graph (4b) are shown here. If the graph is similar to the one on the right, users should follow the troubleshooting guidance in Step 15.

The next important results that can be used as a quality control checkpoint are the graphs generated in Step 18 (methylation calling) (Fig. 5), which provide essential insights into strand specificity and bisulfite conversion efficiency, both of which are crucial for accurate methylation quantification.

**Figure 5:**
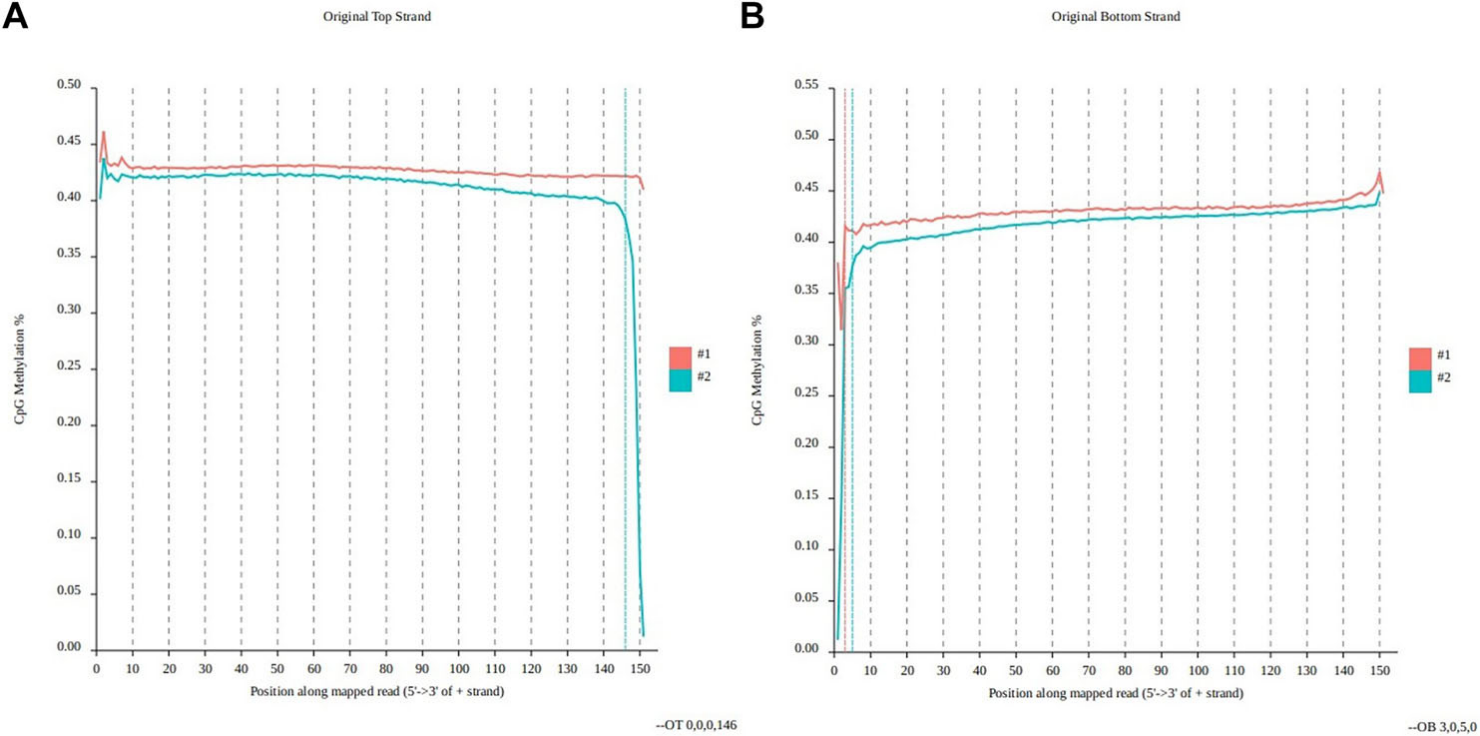
OT (5a) and OB (5b) graphs. Graphs generated in step 18 of methylation calling. #1 forward strands; #2 reverse strands.

If both OT and OB values are zero, or the graph shows a single flat line, it may indicate issues with strand recognition, bisulfite conversion failure (cytosine‐level reports can be generated in step 21), or incorrect alignment settings. In this case, users should refer to the troubleshooting solutions for Step 18 before proceeding.

After completing Steps 19–21, which mark the end of the bash code, users need to migrate to an R environment, such as RStudio, where further methylation analysis will be performed. The first two graphs generated in Steps 37 and 38 (Fig. 6) can also serve as QC checkpoints, as they show how CpG coverage is distributed along the sample sequence and the percentage of CpG methylation.

**Figure 6:**
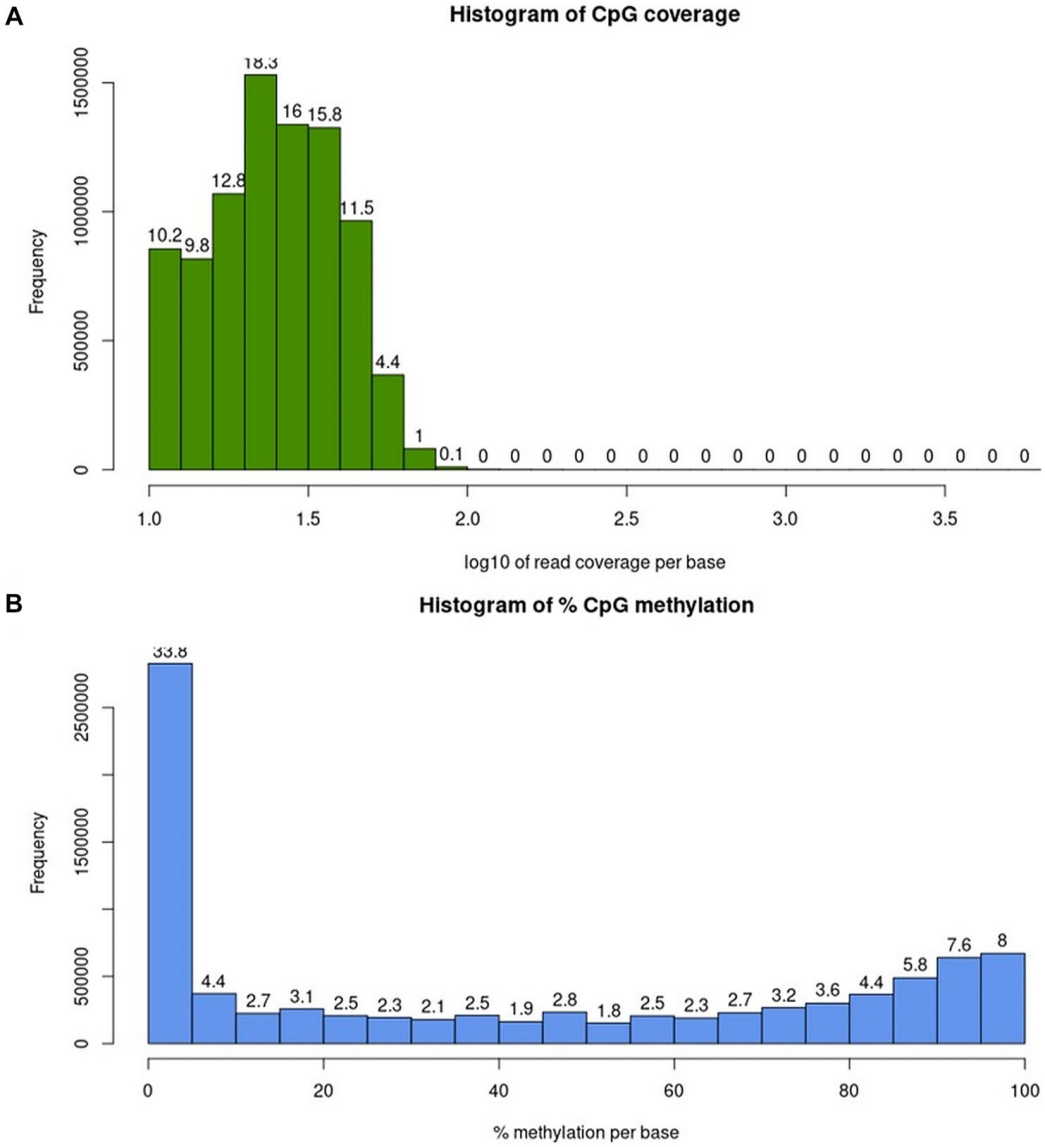
CpG coverage and methylation (%). Graphs were generated after running the code in Steps 37 (6a) and 38 (6b).

With these quality control checkpoints successfully validated, users can confidently proceed with the final analytical stages, including differentially methylated cytosines analysis, sample correlation, principal component analysis, and tiling window analysis. These steps provide a deeper understanding of methylation patterns and their potential biological implications.

## 6 PERFORMANCE BENCHMARKING

To assess the computational efficiency of TNMDW compared to existing pipelines, we benchmarked its performance against Bismark and MethPipe, two widely used tools for DNA methylation sequencing analysis. All pipelines were executed starting from raw paired‐end reads (after trimming) through methylation calling, using a consistent dataset comprising 20 human samples from the Healthy MiNDS (Mother Influences on Child Neurobehavioral Development Study) cohort study (Micali et al., 2025) (10 cases and 10 controls established previously; mean FASTQ file size: 7.82 ± 1.58 GB per sample, with 134M paired‐end reads of 151 bp on average).

Each pipeline was run according to its official documentation, with no modifications to internal parameters beyond those necessary for standardized comparison. All analyses were performed on the same high‐performance Linux workstation, with 20 threads allocated per process to ensure equitable conditions.

We monitored wall‐clock execution time and maximum RAM consumption for each analytical stage: read alignment, duplicate marking, and methylation calling. For each sample, each step was timed independently, and memory usage was recorded. Summary statistics (mean ± standard deviation) are reported in Table 7 for each pipeline and step.

**Table 7:** Comparison of computational performance between TNMDW and existing pipelines

Pipeline	Stages	Software	Time (min)	Peak process memory usage (MB)
	Read alignment	BWA	74.99 ± 26.45	14 ± 1.7
**TNMDW**	Marking duplicates	Picard	33.37 ± 8.25	20.9 ± 14
	Methylation calling	MethylDackel	14.6 ± 3.97	184 ± 8.86
	**Total**		**122.96 ± 38.67**	
	Read alignment	Bismark	889.14 ± 265.16	3.9 ± 0.5
**Bismark**	Marking duplicates	Bismark	32.11 ± 8.77	18.7 ± 6.9
	Methylation calling	Bismark	369.81 ± 92.43	5657.8 ± 981.1
	**Total**		**1291.06 ± 366.35**	
	Read alignment	MethPipe	42.68 ± 8.83	26.1 ± 4.17
**MethPipe**	Marking duplicates	MethPipe	20.21 ± 3.48	4.8 ± 0.2
	Methylation calling	MethPipe	62.78 ± 11.65	7413.9 ± 632.8
	**Total**		**125.67 ± 23.96**	

All stages were performed using 20 samples (10 cases and 10 controls; mean input size: 7.82 ± 1.58 GB per sample; mean 134.2M reads per sample). The hardware specifications used for the comparisons are described in Section 7.1 “Equipment”. Runtime and memory usage may be influenced by differences in internal implementations, parallelization strategies, and parameter configurations across tools. Values should be interpreted as indicative rather than strictly comparable benchmarks. RAM values represent peak process memory usage measured during execution and may not capture memory allocated by subprocesses or I/O operations. Values should be interpreted as indicative comparisons under the tested conditions.

A one‐way ANOVA revealed statistically significant differences in execution time among the three pipelines (F(2, 94.9) = 31.3, *p* < .001). Due to a violation of homogeneity of variance (Levene's test: p < .001), Games‐Howell post hoc tests were conducted for pairwise comparisons. These analyses demonstrated that TNMDW's total runtime was significantly shorter than Bismark's (*p* < 0.001), while no significant difference was observed between TNMDW and MethPipe (*p* = 0.287). This confirms that TNMDW achieves computational efficiency comparable to MethPipe while substantially outperforming Bismark, the most widely used tool for methylation analysis.

We next assessed global methylation concordance across pipelines, revealing high consistency in methylation estimates: Bismark (40.7% ± 6.38), MethPipe (42.0% ± 1.65), and TNMDW (40.7% ± 6.07). A Kruskal‐Wallis test revealed no statistically significant differences in methylation distributions across the three pipelines (χ²(2) = 1.731, *p* = 0.4208). These results confirm that TNMDW provides biologically equivalent methylation calls to established pipelines, while its demonstrated computational advantages establish it as a robust, efficiency‐focused solution for modern DNA methylation analysis.

## 7 DISCUSSION

TNMDW provides a modular, standardized solution for targeted DNA methylation profiling that addresses important challenges related to reproducibility and workflow integration for epigenetic research. Our study, in addition to providing a best‐practices guide to TNMDW with extended QC and statistical steps, shows consistent behavior across analytical stages while maintaining methylation call accuracy comparable to established pipelines.

TNMDW is particularly well‐suited for targeted short‐read bisulfite sequencing, enabling human DNA methylation studies across diverse biological and clinical research contexts (“Analyzing Twist Targeted Methylation Sequencing Data Using the Twist Human Methylome Panel | Twist Bioscience,” accessed July 30, 2025). It can be applied to investigate epigenetic alterations associated with diverse outcomes, including environmental exposures, disease states, and developmental processes. The systematic incorporation of QC steps at critical stages ensures high‐quality data for downstream analyses. This rigorous validation process enhances the reliability of DNA methylation studies, ultimately enabling the generation of biologically meaningful and reproducible insights into epigenetic regulation.

Compared to integrated workflows such as Bismark's integrated tools or MethPipe, TNMDW offers enhanced modularity and flexibility by incorporating widely used and independently maintained tools (e.g., Trim Galore, FastQC, BWA‐meth, and methylKit), which increases both reproducibility and transparency (Gentleman et al., 2004; Krueger & Andrews, 2011). The modular architecture extends beyond human applications to diverse species and experimental designs. Researchers can readily adapt the workflow by swapping reference genomes (e.g., GRCm38 for mouse studies), adjusting tiling window parameters, or incorporating custom R scripts for novel contexts such as plant epigenomics, while preserving the built‐in validation steps. This flexibility is particularly valuable for emerging enzymatic conversion protocols and targeted approaches such as RRBS, where traditional WGBS‐optimized pipelines are often inadequate. By natively supporting both bisulfite and enzymatic methods while integrating downstream analyses (DMC detection, β‐value quantification, and PCA clustering) within a unified R environment, this pipeline eliminates workflow fragmentation and reduces opportunities for human error. These features make it well suited for biomarker discovery in cancer, neurodevelopmental disorders, and environmental epigenetics, where rapid iteration and analytical transparency are paramount.

Our approach complements previous large‐scale efforts, such as those by Gong et al. and the SEQC2 EpiQC consortium, which have focused on benchmarking and evaluating variability across methylation sequencing platforms (Foox et al., 2021; Gong et al., 2022). In contrast, TNMDW emphasizes practical implementation, workflow integration, and reproducibility, providing an end‐to‐end framework tailored to targeted methylation sequencing data.

However, some limitations should be acknowledged. Reliance on command‐line tools requires a moderate level of bioinformatics proficiency and access to computational infrastructure, though detailed troubleshooting guidance helps mitigate this barrier. In addition, the current implementation is optimized for short‐read sequencing data and has not yet been extensively benchmarked with long‐read platforms or single‐cell bisulfite sequencing technologies. Also, benchmarking results should be interpreted with caution, as differences in tool implementation and parallelization strategies may influence performance metrics.

While we focused on benchmarking TNMDW's computational performance against alternative pipelines, we did not directly compare TNMDW with and without the additions proposed in this best practices guide. This is because our enhancements do not alter the core computational steps of the original TNMDW protocol. Instead, they aim to improve usability, reproducibility, and biological interpretability by offering step‐by‐step guidance, troubleshooting for common issues, additional quality control checkpoints, and a built‐in downstream analysis framework in R. These additions are particularly valuable for users with limited bioinformatics support and for projects that require clear documentation and publication‐ready outputs. While they do not significantly impact processing time, they substantially enhance the analytical robustness and transparency of DNA methylation studies based on TNMDW.

Taken together, TNMDW addresses a critical need in the field by providing a scalable, transparent, and adaptable protocol for analyzing targeted DNA methylation data. Its emphasis on modularity, reproducibility, and user control makes it a valuable tool for epigenetic studies seeking both analytical depth and methodological rigor. By extending the original TNMDW protocol with a comprehensive suite of improvements, from enriched documentation to integrated statistical analyses, this guide helps researchers to generate high‐quality, interpretable results with greater confidence. We believe that these contributions support ongoing efforts to standardize and improve the accessibility of targeted methylation sequencing data analysis.

## 8 MATERIALS

### 8.1 Equipment

The computer hardware settings used were as follows: Processor: Intel® Core™ i9‐7980XE CPU @ 2.60 GHz × 36; Memory: 128 GB; Disk capacity: 21 TB; Operating System (OS): Ubuntu 22.04.5 LTS; OS type: 64‐bit. This configuration was used to process datasets with up to 193 samples. For smaller datasets, the pipeline can be executed on machines with lower computational capacity by adjusting thread usage and memory allocation accordingly.

### 8.2 Input data

All data/files required as input at each stage of the pipeline are presented in Table 3. All scripts and pipeline configurations used in this protocol are available at https://github.com/PGT‐Lab/TNMDW‐best‐practices. Example datasets and reference genomes are available upon request. Publicly available DNA methylation sequencing datasets can also be obtained from repositories such as GEO to reproduce and test the workflow. For example, WGBS datasets from autism spectrum disorder studies, such as SRR36669993 (female) and SRR36693388 (male) from BioProject PRJNA1397483, can be used as representative test cases for benchmarking and validating the pipeline.

### 8.3 Study dataset and biospecimens

The sequencing dataset used in this study was obtained from DNA samples isolated from participants in the ‘Healthy’ MiNDS cohort, which investigates the intergenerational effects of maternal adverse childhood experiences on child neurodevelopment. Case and control group categorizations were previously defined within the original Healthy MiNDS study design (Micali et al., 2025).

Placental tissue was collected within 10 min after delivery, sampled from the fetal side following removal of the amniotic membrane. To ensure broad coverage of DNA methylation patterns, the placenta was divided into four quadrants, from which two ∼0.1 cm³ chorionic villi fragments were obtained per quadrant using sterile forceps and scalpel.

Each sample was washed in sterile 1× PBS and stored in AllProtect Tissue Reagent (Qiagen, USA) at 4°C for 24 hr, then transferred to −20°C until processing. For DNA extraction, small tissue fragments (7–8.5 mg) from each quadrant were pooled, yielding 28–34 mg of tissue per sample. DNA was extracted using the AllPrep DNA/RNA Mini Kit (Qiagen, USA), following the manufacturer's protocol (“AllPrep DNA/RNA Mini Handbook ‐ QIAGEN,” n.d.). The extracted DNA had a mean concentration of 461.81 ng/µL and quality metrics consistent with high‐purity DNA.

Targeted methylation profiling was performed using the NEBNext® EM‐seq™ Kit combined with the Twist Targeted Methylation Sequencing platform (Twist Bioscience, USA), following the manufacturer's instructions (New England Biolabs, 2025; “Twist Targeted Methylation Sequencing Protocol | Twist Bioscience,” accessed July 30, 2025). This method employs enzymatic methylation conversion rather than bisulfite treatment, minimizing DNA degradation and yielding higher‐complexity libraries. Libraries were sequenced on the Illumina NovaSeq X Plus platform. The Twist panel covers approximately 3.98 million CpG sites across 123 Mb of the genome, focusing on functionally relevant methylation regions (“Twist Human Methylome Panel | Twist Bioscience,” accessed July 30, 2025).

### 8.5 Funding

This study was mainly funded by the National Institute of Mental Health (NIMH; R01 MH121070‐01A1) and Fundação de Amparo à Pesquisa do Estado de São Paulo (FAPESP; Research Support Foundation of the State of São Paulo; 2019/21612‐0, 2021/04819‐0, and 2025/02111‐1), as part of a partnership between these agencies. The study was also supported by the Conselho Nacional de Desenvolvimento Científico e Tecnológico (CNPq; National Council for Scientific and Technological Development; grant numbers 427215/2018‐8 and 306982/2021‐8) and Coordenação de Aperfeiçoamento de Pessoal de Nível Superior (CAPES—Finance Code 001). CMC was supported by Fundação de Amparo à Pesquisa do Estado de São Paulo (FAPESP number: 2024/14539‐3) and by the National Council for Scientific and Technological Development (CNPq) fellowship #177188/2023‐5.

### 8.6 Author Contributions

**Vitória Rodrigues Guimarães Alves**: Conceptualization; writing—original draft; methodology; validation; software; formal analysis; data curation; investigation. **Sheila T. Nagamatsu**: Conceptualization; investigation; writing—review and editing; methodology; validation; software; formal analysis; data curation. **Vanessa Kiyomi Ota**: Conceptualization; investigation; writing—review and editing; validation; methodology; software; formal analysis; data curation. **Cibele Edom Bandeira**: Conceptualization; investigation; writing—review and editing; validation; methodology. **Janitza L. Montalvo‐Ortiz**: Writing—review and editing. **Danilo Micali**: Writing—review and editing. **Ana Carolina Coelho Milani**: Writing ‐ review and editing. **Ivaldo Silva**: Writing—review and editing. **Cristiane Seixas Duarte**: Writing—review and editing. **Andrea Parolin Jackowski**: Writing—review and editing. **Jonathan Posner**: Writing—review and editing. **Carolina Muniz Carvalho**: Conceptualization; investigation; writing—review and editing. **Sintia Iole Belangero**: Conceptualization; writing—review and editing; supervision; project administration; investigation.

### 8.7 Conflict of Interest

The authors declare no conflict of interest.

## Data Availability

The data that support the protocol were stated in the manuscript. Raw sequencing data from this protocol are available from the corresponding author (S.I.B.) upon reasonable request, as they are part of the ‘Healthy’ MiNDS cohort study (Micali et al., 2025).
